# Identity crisis: exploring the boundaries of cell type identification in the age of single-cell transcriptomics

**DOI:** 10.3389/fncel.2026.1819116

**Published:** 2026-05-05

**Authors:** Seulkee Yang, Sudeeksha Tyagi, Christian Rosenmund, Melissa A. Herman

**Affiliations:** Institute for Neurophysiology, Charité Universitätsmedizin—Berlin, Berlin, Germany

**Keywords:** single-cell transcriptomics, cell identity classification, cortical neuronal identity, *in vitro* vs *in vivo*, primary neuronal cell culture

## Abstract

The rise of single-cell transcriptomics and comprehensive reference atlases promised a unifying molecular framework to classify cell identity. Yet transcriptomic identities are often interpreted outside the environmental contexts in which they arise. Here, we analyzed primary cortical cultures, which lack native tissue architecture, to compare their transcriptional profiles to multiple *in vivo* mouse cortical reference datasets. We found that while core molecular signatures for major neuronal subclasses are largely preserved *in vitro*, the loss of *in vivo* structure triggers high transcriptional divergence associated with metabolic and physiological state. We also identified clusters that consistently show low confidence in the classification tool. These ambiguous populations express incomplete canonical marker profiles resulting from a lack of structural cues necessary for full maturation. These observations suggest that while transcriptomic reference frameworks capture major aspects of neuronal identity, their interpretation can become less certain when cells are profiled outside their native environment. Our findings highlight the importance of considering environmental context when interpreting transcriptome-based cell type annotations and provide a resource for understanding how neuronal transcriptional programs are reshaped *in vitro*.

## The scientific necessity of cell type classification

Cells are the fundamental unit of life. To better understand their roles at the organismal level, cell types are differentiated through structural and functional characteristics. In neuroscience, this cell type classification has historically relied on disentangling cell identity across various dimensions, such as morphological, anatomical, functional, and developmental lineage-based criteria (Zeng and Sanes, 2017).

Early cell type identification relied on morphological and anatomical classification of neurons. For instance, the illustrations of Ramón y Cajal revealed a substantial neuronal diversity across species and brain regions (Garcia-Lopez et al., 2010). Yet, electrophysiology and connectomics techniques were critical for providing functional dimensions to cell type classification (Cole and Curtis, 1939; Hodgkin and Huxley, 1939; Nassi et al., 2015; Kornfeld and Denk, 2018). Electrophysiology provides a framework for classifying neurons based on their biophysical attributes, such as membrane conductance, firing thresholds, and temporal firing patterns (Contreras, 2004), while connectomics expands this perspective by examining how neurons integrate and interface at the circuit level. Mapping the brain’s network architecture has marked a significant development in the field of neuroscience, with comprehensive connectomic datasets providing structural and functional insights into neural circuits (Sporns, 2011; Oh et al., 2014; Turner et al., 2022; Liu et al., 2025).

Spatial context and developmental lineage add another dimension to cell type classification. Cells occupying specific anatomical layers or regions often adopt specialized roles (Greig et al., 2013; Holguera and Desplan, 2018). In addition, lineage-tracing studies have shown that cells arising from distinct progenitor zones follow divergent differentiation paths, acquiring different structural and functional traits (Kretzschmar and Watt, 2012; Ma et al., 2018). In this landscape, as our understanding of neuronal identity becomes increasingly multifaceted, it is important to consolidate these distinct properties of the cells into their identity. It has become evident that these different features each capture disparate facets of neuronal identity, without necessarily converging into a singular, stable definition of ‘cell type’.

In view of these complexities, transcriptomics emerged as a promising strategy to construct a unifying molecular framework for cell type classification (Trapnell, 2015; Poulin et al., 2016; Yuste et al., 2020; Zhang et al., 2023). By unravelling the molecular code of cells that orchestrates different developmental, spatial, morphological, and functional aspects, transcriptomics offers a bottom-up outlook into cell identity. This was significantly expedited by the advent of single-cell transcriptomic profiling, first demonstrated by Tang et al. (2009), which presented a strategy for analyzing and classifying individual cells by applying their most basic defining properties.

## The promise and limit of transcriptomic cell identity

Single-cell RNA sequencing methods yield a substantial and extensive dataset (Kharchenko, 2021). An inevitable repercussion of this scale is the curse of dimensionality and loss of interpretability (Imoto et al., 2022). To address this, statistical frameworks and dimensionality reduction methods emerged as essential tools to prune noisy data and extract a meaningful subset of biologically relevant information (Van der Maaten and Hinton, 2008; McInnes et al., 2018). Cell type annotation is performed on these reduced-dimensional embeddings, traditionally through the identification of marker genes (Pasquini et al., 2021). However, this manual approach is inherently subjective and labor-intensive. To address these limitations, a growing number of automated cell type annotation methods have emerged (Li et al., 2025). However, despite their efficiency, the reliability and interpretability of such automated annotations, especially in contexts where the reference may not capture the full diversity or novelty of the query data, remain areas of active investigation.

With the advancement and increase in the accessibility of single-cell transcriptomic techniques, a multitude of studies now aim at deciphering cell identity through transcriptomic profiles of neurons in different brain regions. In the primary visual cortex, for instance, from 2016 to 2018, the number of transcriptionally identified glutamatergic neuron subtypes rose from 19 to 33 (Tasic et al., 2016; Tasic et al., 2018). These studies highlight the fast-evolving nature of the field; however, they also allude to a certain disarray in our current understanding of transcriptomic cell types. While common cell types have been identified across different regions, to what extent is the molecular identity of a neuron anchored to its functional and anatomical architecture? In fact, it has been observed that transcriptomic clusters can also fail to distinguish neurons with distinct projection patterns or, therefore, seemingly distinct roles (Mao and Staiger, 2024; Kim et al., 2020).

Transcriptomic profiling has become a widely applied framework for classifying cells and identifying novel cell types. However, single-cell transcriptomes capture both relatively stable cell-type-associated genes and context-dependent state genes reflecting metabolic activity, stress responses, or environmental adaptation. Changes in the balance between these programs may influence how confidently cells can be assigned to reference-defined cell types. In this article, we explore the nuances of transcriptomic cell type classification using single-nucleus RNA sequencing (snRNA-seq) data from dissociated primary cortical cultures. This *in vitro* system imparts a unique outlook into the transcriptomic profiles of cells stripped of anatomical architecture and spatial cues. When a cell is dissociated from its natural environment and grown in a dish, it experiences altered signaling landscapes, connectivity, and metabolic conditions. These changes raise an important question- how much of the transcriptomic identities defined *in vivo* remain detectable under such conditions? By comparing cultured cortical neurons to *in vivo* reference atlases across multiple developmental stages, we examine how well transcriptome-based cell-type annotations translate across environments. Thereby, we aim to assess which aspects of neuronal transcriptional identity remain robust *in vitro* and which features diverge under an altered environmental context.

## Global molecular signatures are preserved in cultured cortical neurons

To examine the influence of spatial and network contexts on neuronal identity, we profiled cortical neurons cultured *in vitro* using snRNA-seq. The primary cultures were generated from cortices dissected from early postnatal days (P) 0–2 pups and grown *in vitro* for 14 days before being processed for snRNA-seq. Following quality control, we generated four separate *in vivo* reference models using publicly available snRNA-seq datasets from mouse visual cortex at P8, P14, P17, and P21 (Cheng et al., 2022). These references were selected due to their comparable sequencing resolution and span of developmental timepoints, which was imperative not only to understand cell-type composition but to establish the maturity and developmental background of cultured neurons. To evaluate the robustness of this annotation framework independently of our dataset, we first performed a self-validation using *in vivo* reference cells with known ‘actual’ labels. Label transfer showed high agreement with the original annotations, with a strong confusion matrix performance, high F1 scores, and true positive rates at the subclass level (Supplementary Figure S1A). Type-level annotation was also feasible, although performance was modestly reduced relative to subclass-level mapping (Supplementary Figure S1B).

**Figure 1:**
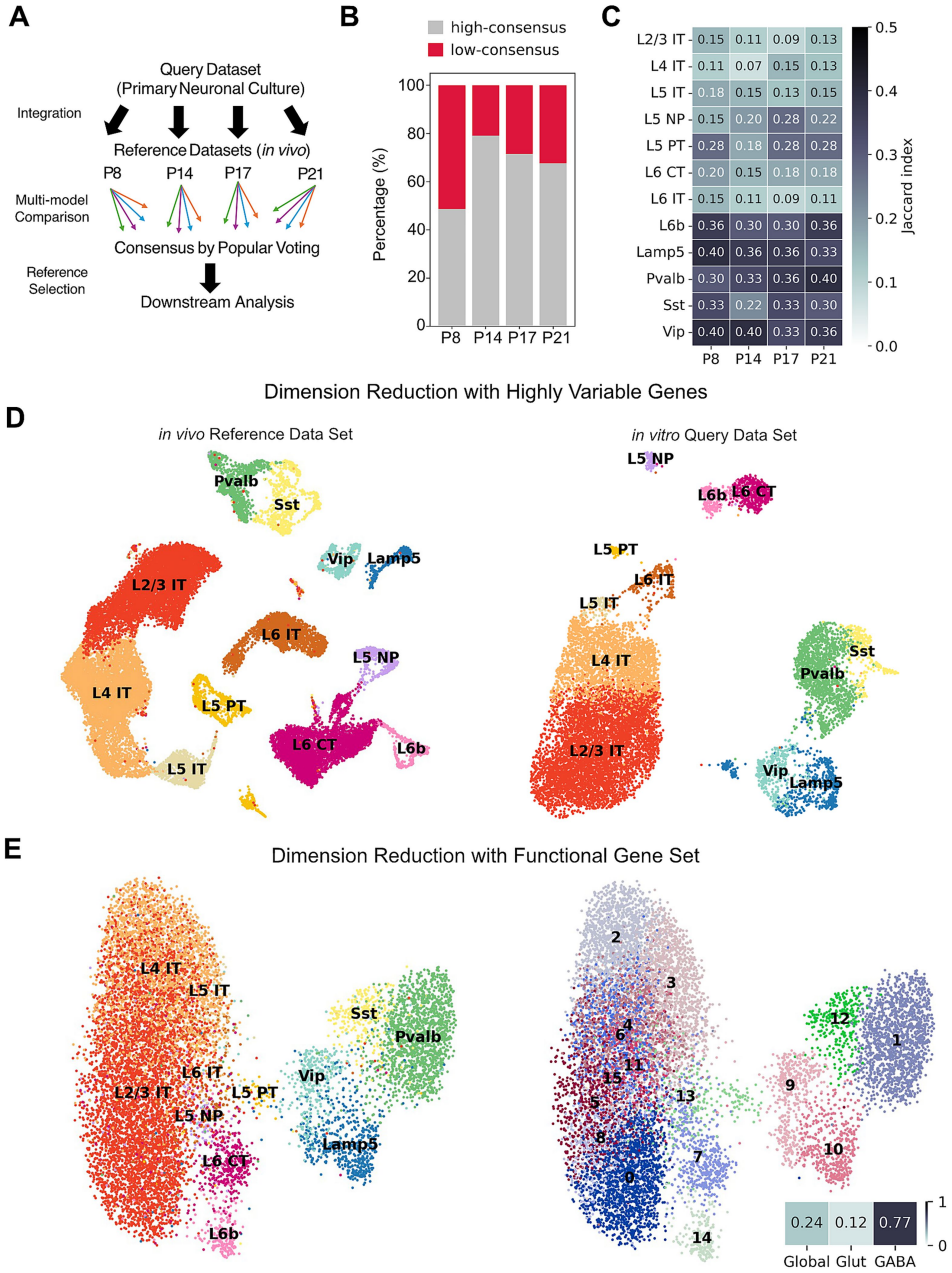
Global molecular signatures of cell identity are preserved in cultured cortical neurons. **(A)** Scheme of workflow to assign cell subclass annotations for a query dataset by using multiple reference sets using the PopV framework, which integrates predictions from eight automated annotation methods across multiple reference datasets. **(B)** Stacked bar plot showing the proportion of cells assigned to high-consensus (grey) or low-consensus (red) Leiden clusters when mapping *in vitro*-derived neurons to postnatal mouse cortical reference atlases (P8, P14, P17, and P21) at subclass-level. Cell-level agreement was defined by the number of annotation models supporting the majority-vote label. Clusters with fewer than 30% cells supported by five or fewer of eight models were classified as high-consensus. **(C)** Heatmap showing the Jaccard index of overlap between the top marker genes (*n* = 30 per subclass, identified by differential expression) for *in vitro*-derived neuronal subclasses and *in vivo* cortical references at postnatal stages P8, P14, P17, and P21. Higher Jaccard indices indicate greater similarity in subclass-specific transcriptional programs. Color scale ranges from 0 to 0.5. **(D)** UMAP visualization of the P14 cortical reference (left), and of the *in vitro* dataset alone (right). Only high-consensus cells were included. Cells are colored by annotated neuronal subclass. **(E)** UMAP embeddings of *in vitro* cortical neurons using a curated 172-gene ion channel panel (GO:0005244). Cells are colored by PopV subclass annotations derived from 4,000 highly variable genes (left) or by Leiden clusters (right). Agreement between ion channel-based clusters and PopV subclass labels was quantified using the ARI. At the whole-population level, concordance was modest (Global ARI = 0.24), with very low agreement among glutamatergic subclasses (Glut = 0.12) but high concordance within GABAergic subclasses (GABA = 0.77).

Traditional clustering and annotation approaches can be limited by dimensionality reduction artifacts, parameter sensitivity, and a lack of standardization. To address these challenges and robustly map our cultured cells to *in vivo* cortical types, we used Popular Vote (popV), an automated cell-type annotation framework that integrates eight different automated annotation methods to assign consensus-based cell-type labels (Ergen et al., 2024; Figure 1A). We evaluated annotation performance at both subclass and finer type resolution. As type-level annotations consistently yielded lower consensus rates than subclass-level annotations, we used subclass labels for downstream comparison (Supplementary Figure S1C). To assess how well *in vitro* neurons mapped onto *in vivo* identities, we defined a cell-level agreement score as the number of annotation models supporting the majority-vote label. We then summarized annotation confidence at the cluster level by calculating the fraction of cells with support from five or fewer models in each Leiden cluster (Traag et al., 2019). Clusters with fewer than 30% of such cells were considered high-consensus. Across all references, a substantial fraction of cultured neurons achieved high-consensus labels. The highest percentage of confidently mapped cells was obtained with the P14 reference (80%), suggesting that cultured neurons most closely resemble the transcriptional states of the P14 reference dataset (Figure 1B).

To quantify the correspondence between *in vitro* and *in vivo* cell identities beyond annotation labels, we computed the Jaccard index based on the overlap of marker genes between matched cell types across all the developmental stages (Figure 1C). Overall, transcriptomic similarity between *in vitro* and *in vivo* neurons remained broadly comparable, with only modest shifts in Jaccard indices across time points. Excitatory subclasses displayed more variability and lower indices (0.07–0.28) except for L6b neuron (0.3–0.36), indicating reduced separation of laminar- and projection-specific programs *in vitro.* In contrast, inhibitory neurons such as parvalbumin (Pvalb), somatostatin (Sst), Lamp5, and vasoactive intestinal peptide (Vip) maintained higher Jaccard indices (0.22–0.4) with a trend toward stronger similarity at later postnatal stages. We also computed Spearman correlations between matched *in vitro* and *in vivo* subclasses. Global transcriptomic similarity was comparable across developmental stages (Supplementary Figure S1D). Consequently, we used P14 as the baseline reference for interpreting cell identity and divergence in subsequent analyses. Low-dimensional embeddings of the cultured dataset showed clear segregation between excitatory and inhibitory populations, with further separation into canonical laminar and subclass-associated identities, broadly mirroring the organization observed in *in vivo* reference datasets (Figure 1D). Through popV annotation, we established that all major neuronal subclasses identified in the *in vivo* references are retained in our *in vitro* dataset.

To characterize neuronal identity with a biophysical and functional perspective, we restricted the analysis to a set of 172 ion channel genes (Reva et al., 2025, GO:0005244). Despite representing a limited functional program, ion channel-based Leiden clustering overlaid on the transcriptome-wide subclass labels recovered a clear separation between inhibitory and excitatory populations and partially resolved major interneuron subclasses (Vip, Sst, Pvalb, Lamp5), indicating that ion channel expression captures partial information related to neuronal subclass-relevant structure (Figure 1E). To quantify how well ion channel-based clustering aligned with transcriptome-wide identity assignments, we computed the Adjusted Rand Index (ARI) between Leiden clusters derived from ion channel genes only and popV-predicted cell-type labels. Notably, inhibitory (GABAergic) neurons exhibited a markedly higher ARI (0.77) compared to excitatory (glutamatergic) neurons, which showed substantially lower agreement (ARI = 0.12). This observation is consistent with the functional specialization of inhibitory neuronal subtypes, which are defined by distinct firing properties and therefore require tightly regulated and subtype-specific ion channel compositions.

## Context-dependent divergence of transcriptomic profiles from *in vitro* primary neurons

**Figure 2:**
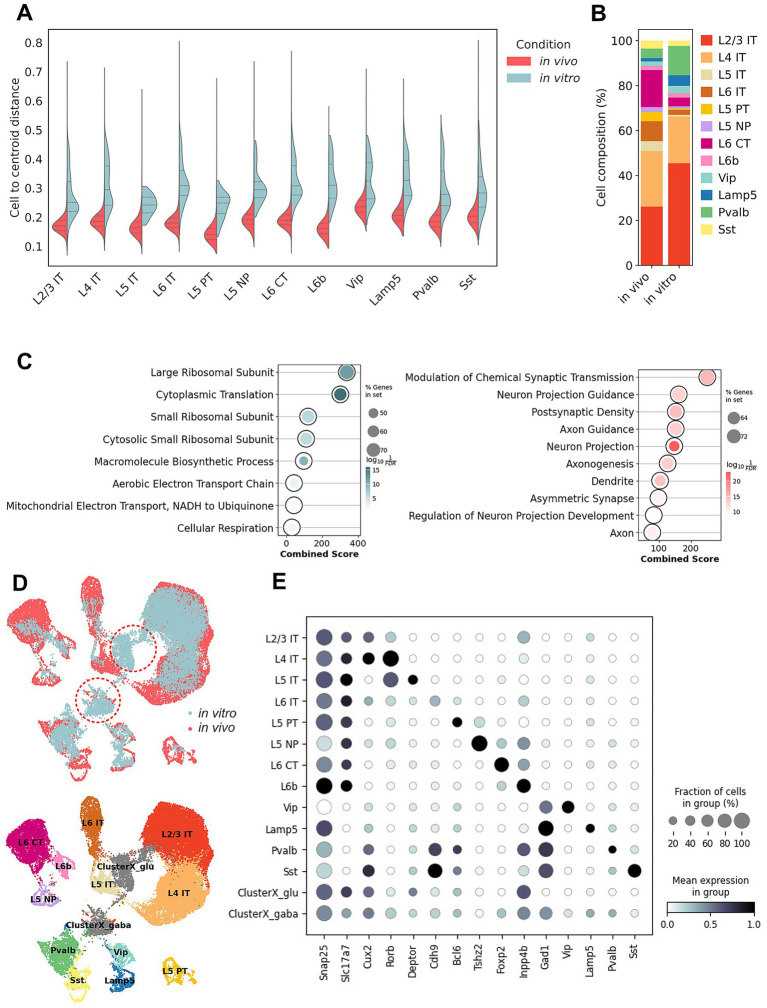
Divergent transcriptional programs in cultured neurons and the emergence of low-confidence neuronal populations. **(A)** Split violin plots showing the distribution of the correlation distance of individual cells to the mean of expression profile of their assigned subclasses within the same biological sample. Distances were calculated for matched neuronal subclasses in *in vivo* (pink) and *in vitro* (teal) conditions. Quartile lines are shown within each violin. Greater distances indicate broader within subclass transcriptional dispersion. **(B)** Stacked bar chart showing the relative abundance of neuronal subclasses *in vivo* and *in vitro* samples. Each color represents a transcriptomically defined subclass. **(C)** Dotplot showing gene ontology enrichment of genes upregulated *in vitro* (left) and *in vivo* (right). **(D)** UMAP embedding of cultured cortical neurons mapped to the P14 reference, colored by batch (up) and popV consensus-based annotation (down). Most cells align with canonical excitatory and inhibitory subclasses, whereas a distinct low-consensus population (defined as Leiden clusters containing more than 30% cells with support from <6/8 annotation models agreeing) forms a separate cluster that cannot be confidently assigned to any *in vivo* cortical type (in red dash). **(E)** Dot plot showing scaled expression of pan-neuronal markers (Snap25), excitatory marker (Slc17a7), GABAergic marker (Gad1), layer- and projection-associated excitatory markers (Cux2, Rorb, Deptor, Cdh9, Bcl6, Tshz2, Foxp2, Inpp4b), and inhibitory subclass markers (Vip, Lamp5, Pvalb, Sst) across cortical subclasses and the two unaligned populations, ClusterX_glu and ClusterX_gaba.

While neurons cultured *in vitro* maintain core transcriptional features of neuronal identity, we wanted to examine the transcriptional divergence caused by the reshaping of environmental cues in culture. We quantified transcriptional dispersion per subclass by measuring the correlation distance of each cell to the mean expression profile of its assigned subclass within each biological sample. *In vitro* cells showed broader distance distributions than their *in vivo* counterparts, indicating increased transcriptional dispersion under culture conditions (Figure 2A). It should be noted that the *in vitro* dataset exhibits overall lower sequencing depth, fewer detected genes, and higher mitochondrial read fractions compared to the *in vivo* references (Supplementary Figure S2). While these differences are expected given the distinct nature of primary cultures versus intact tissue, they represent an additional source of variability that may influence reference-based annotation and the interpretation of transcriptional divergence. Comparison of subclass composition revealed substantial shifts between the *in vivo* and *in vitro* populations (Figure 2B). Deep-layer glutamatergic neurons were notably reduced in culture conditions, whereas upper-layer intratelencephalic (IT) pyramidal neurons were observed at higher numbers. In contrast, *γ*-aminobutyric acid (GABA)-ergic neurons are globally more abundant *in vitro*, with a particularly pronounced expansion of the Pvalb population. To characterize the molecular programs underlying these differences, we identified differentially expressed genes (DEGs) across all neuronal subclasses (Supplementary Figure S3). Gene Ontology analyses revealed a striking separation between pathways preferentially active in cultured versus *in vivo* neurons (Figure 2C). Neurons maintained *in vitro* showed strong enrichment for ribosomal biogenesis, oxidative phosphorylation, and biosynthetic pathways, indicative of increased translational demand and heightened metabolic activity. These signatures likely reflect transcriptional programs associated with cellular state in the artificial culture conditions, including altered nutrient availability, growth factor exposure, and the absence of physiological circuit constraints. In contrast, *in vivo* neurons prominently enriched gene sets associated with synaptic transmission, neurotransmitter release, calcium-dependent exocytosis, and trans-synaptic signaling. These pathways are commonly associated with synaptic communication in intact cortical circuits. Hence, while *in vitro* neurons maintain core transcriptional signatures, their transcriptomes also show enrichment of context-specific gene programs.

Despite the overall transcriptional resemblance of cultured neurons to mid-postnatal cortical stages, our analysis revealed the emergence of a distinct cluster that did not align confidently with any *in vivo* reference population. This cluster consistently formed a distinct group in the low dimensional embedding and showed low consensus scores in popV-based annotations, indicating a lack of clear correspondence to known cortical *in vivo* subclasses (Figure 2D). We assigned two unaligned populations as ClusterX_glu and ClusterX_gaba, which occupy distinct spaces in between the canonical excitatory and inhibitory clusters (Figure 2D). To further investigate the nature of the ambiguous low-consensus cluster, we examined the expression of canonical excitatory and inhibitory marker genes across all subclasses, including ClusterX_glu and ClusterX_gaba (Figure 2E). These clusters showed reduced expression of canonical excitatory and inhibitory marker genes compared to their *in vivo*-aligned counterparts. One possible explanation is that these populations are dominated by state-associated transcriptional programs that reduce the relative prominence of canonical type-defining marker genes used by reference-based annotation methods. Consistent with this interpretation, *in vitro* neurons showed broader transcriptional dispersion within assigned subclasses than their *in vivo* counterparts, indicating an expansion of transcriptional state space under culture conditions. Under such conditions, neurons may retain broad excitatory or inhibitory identity while exhibiting transcriptional profiles that map poorly to existing *in vivo* reference subclasses.

## Discussion

Since Robert Hooke observed the thin slice of cork under the microscope and discovered cells, biologists have sought to characterize and classify them. These efforts have been crucial, not only for organizing the complexity of biological systems but also for understanding the developmental and functional trajectories of specific cell types. This dates back to the early 1900s, when growing disputes and discussions about neuronal structure and function pushed the field to develop tools that could isolate and characterize single cells. This culminated in the prominent advancement in the field by Ross Harrison, who developed the first *in vitro* neuronal culture (Harrison, 1906). He noted that this approach offered the key advantage of allowing the study of individual cells independent of environmental and spatial influences. Now, in a time defined by vast datasets and high-resolution cellular profiling, our study took advantage of this reductionist approach to examine how much of neuronal transcriptional identity is preserved when cells are removed from their native environment.

Our findings show that cortical neurons cultured for 14 days from P0-P2 brains show transcriptomic profiles most closely aligning with P14 reference data. Additionally, the neurons maintain core cellular heterogeneity, including excitatory glutamatergic and inhibitory GABAergic subclasses. This suggests that key aspects of maturation can unfold *in vitro*, even without the *in vivo* spatial and complex network context, indicating that several major transcriptional features of neuronal subclasses remain identifiable *in vitro*. The inhibitory neurons particularly display a stronger correspondence to *in vivo* transcriptional signatures, whereas transcriptional separation between excitatory subclasses weakens *in vitro*, with reduced marker gene coherence and increased cluster overlap. This suggests that excitatory identities are more sensitive to extrinsic environmental cue or network context that are absent in culture. Additionally, it may be that transcriptional differentiation between excitatory subtypes is more subtle, and more easily masked by *in vitro*-specific changes in gene expression. For instance, a recent multi-modal cell characterization of excitatory neurons from human cortex found that position in cortical layer plays a strong role in distinguishing subtype classes (Dalley et al., 2026). The differential stability across classes implies that use of transcriptomic clustering to define identity may be especially fragile for excitatory neurons whose phenotypes depend on network context, while inhibitory neuron types exhibit greater transcriptional autonomy.

The emergence of an extra cluster *in vitro*, disjointed from any established *in vivo* cortical identities, highlights the difficulty of mapping some *in vitro* transcriptional profiles onto existing *in vivo* reference identities. Using gene-set enrichment analyses, we demonstrated that the transcriptome mirrors the physiological properties dictated by the environment- metabolic and biosynthetic transcriptional programs enriched in *in vitro*, whereas the *in vivo* environment drives the expression of genes essential for synaptic cellular communication. Consistent with this, Baumann et al. (2025) recently reported clusters of transcriptionally undefined cells *in vitro* and demonstrated that this molecular divergence is driven by extrinsic cues. Notably, they also found that excitatory neurons were disproportionately affected by these context-dependent transcriptional changes. Together, these observations suggest that single-cell transcriptomes capture both cell-type-associated programs and context-dependent state-associated gene expression. Hence, transcriptomic marker expression should be interpreted in the context of cellular state and environment. Notably, reliable identification of cell-type-specific markers has been shown to require validation across multiple datasets, at least five, according to Fischer and Gillis (2021).

Waddington painted a landscape, an analogy for cell fate determination, of a ball rolling down a branching slope onto valleys, which represent stable differentiated cell states (Waddington, 2014). However, given the current understanding of cell identity, it is essential to look at these stable cell fates as high-dimensional attractor states in a gene regulatory landscape (Huang et al., 2005). This perspective implies that plastic and context-dependent attractor states may shift under altered environmental conditions. Although previously studied in developmental settings, our findings reveal that differentiated postnatal neurons *in vitro* can also display plastic transcriptional profiles. This underscores the need to evaluate whether observed transcriptomic differences correspond to altered cellular function or represent adaptive, context-dependent states.

A recent study elegantly demonstrated that a small subset of genes, particularly those encoding ion channels, is sufficient to capture the identity and variability of neuronal subtypes (Reva et al., 2025). Similarly, our results show that, while the *in vitro* system does not fully recapitulate global transcriptomic signatures and exhibits some culture-specific expression biases, the key functional transcriptional features of neurons appear to be preserved. This concept aligns with studies integrating multimodal datasets where morphoelectric characteristics of cortical GABAergic cells were integrated with their transcriptomic profile (Gouwens et al., 2020). The study found 28 cell types with the three integrated features in contrast to the previously described 60 ‘transcriptomic-types’. The study by the NIH’s BRAIN Initiative Cell Census Network (BICCN) also demonstrated how integrating morphology, electrophysiology, and spatial context can refine our understanding of neuronal diversity beyond transcriptomic labels alone (BICCN, 2021).

Transcriptomics provides a powerful lens to define a cell through its fundamental molecular signatures; yet, these signatures are inherently complex and transient. Our findings suggest that key molecular signatures are robust and can emerge even under simplified spatial and network contexts. Nevertheless, these observations underscore the importance of reframing neuronal identity, not solely as a fixed transcriptional entity defined by a molecular signature but as a dynamic, context-dependent state shaped by both intrinsic programs and extrinsic environments (Morris, 2019). As data grows denser and algorithms become more opaque, there is a growing need for unified and concerted analytical frameworks, particularly for cell type annotation. A multimodal approach not only reflects the full complexity of biological identity but also helps disentangle genuine biological transitions from artifacts introduced by analytic frameworks. Defining a cell type is not a matter of assigning it to a fixed transcriptional category but of understanding the regulatory, spatial, and functional conditions under which cell states emerge and stabilize.

## Methods in brief

All animal experiments were performed in accordance with institutional and local authorities, as permitted by Landesamt für Gesundheit und Soziales (LaGeSo) Berlin under license numbers T0220/09.

Neurons were dissociated from P0-P2 male C56/BL6N mouse cortex and maintained for 14 days *in vitro* (DIV14), allowing us to observe transcriptional changes that emerge outside the native brain environment. Frozen cultures were processed to undergo single-nucleus encapsulation, barcoding, and sequencing library preparation using the Chromium GEM 3’ Kit v3.1 from 10X Genomics, and the sequencing was performed on a NovaSeq X Plus sequencer (Illumina).

Raw reads were processed using Cell Ranger v9.0.1 and aligned to the GRCm29 (2024-A) mouse reference to generate the gene expression count matrix. Ambient RNA was removed using CellBender (Fleming et al., 2023). The subsequent analysis was performed using SCANPY (Wolf et al., 2018). After removing doublets (Gayoso and Shor, 2022) and low-quality cells based on standard QC metrics (n_genes, n_counts, and mitochondrial content; Supplementary Figure S2), a total of 14,723 cells and 26,253 genes were retained for analysis.

Detailed Methods can be found in the Supplementary Materials section.

## Data Availability

Publicly available datasets were analyzed in this study. Raw and processed snRNA-seq data reported in this study are publicly available on the Zenodo repository at Doi: 10.5281/zenodo.19554693.
