# Supplementary material for: Identity crisis: exploring the boundaries of cell type identification in the age of single-cell transcriptomics
Source: Front Cell Neurosci. 2026 May 5;20:1819116. doi: 10.3389/fncel.2026.1819116 (PMC13183566; doi:10.3389/fncel.2026.1819116)
Supplement: Supplementary file 1 [file Data_Sheet_1.pdf]

# Supplementary Materials for Yang et al., 2026

# Supplementary Methods

All animal experiments were performed in accordance with institutional and local authorities, as permitted by Landesamt für Gesundheit und Soziales (LaGeSo) Berlin under license numbers T0220/09.

*Psuedo-Banker Primary Neuronal Culture*

Three weeks prior to plating primary neurons, astrocyte cultures for feeder layer were prepared. In brief, cortices were dissected from postnatal day 0-2 (P0-P2) wildtype (C57/BL6N) mice of either sex and digested for 20 min in Trypsin-EDTA solution (0.05%; Gibco; REF 25-300-054) at 37°C. After 20 min, Trypsin-EDTA solution was replaced with DMEM (Gibco; REF 31-966) supplemented with 10% fetal calf serum (Gibco; REF 26-140), and penicillin/streptomycin (10 U/mL/1 µg/mL; Roche; Cat. No. 11-074-440-001). Cortical tissue was mechanically dissociated by pipetting, and the cell solution was split between two T75 culture flasks each containing 13 mL of supplemented DMEM. Astrocytes were grown for two weeks in a 37°C (5% $CO_2$) incubator, with media change on DIV3 and DIV10.

At DIV13, supplemented DMEM was removed from the T75 culture flasks (Falcon; REF 353136) and cells were incubated with trypsin for 1 min on the sterile bench. Trypsin was then removed, and flasks were returned to the 37°C (5% $CO_2$) incubator for a further 6 min incubation without solution. Cell adherence was then disrupted by mechanical force against the flask, followed by serological pipetting with 7 mL of supplemented DMEM. The cell solution was then plated in supplemented DMEM into 6-well Multiwell Tissue Culture Plate (Falcon; REF 353046) with pre-made paraffin posts at a density of ~20 cells/$mm^2$ and grown to confluence (~5-7 days).

On the day of primary neuron culture, 25 mm coverslips previously coated with a mixed Poly-D-lysine (0.1 mg/mL; Sigma Cat. No. P6407) and Collagen I (~0.1 mg/mL; Gibco REF A10483-01) solution (10 mM acetic acid) were incubated in sterile DPBS (Gibco; REF 14190-094) for 1-2 hours at 37°C (5% CO2). After incubation, DPBS was removed and 250 µL volume of sterile Poly-L-ornithine (Sigma; Cat. No. P4957) to the center of the coverslip, held by surface tension and incubated for at least 1 hr at 37°C (5% $CO_2$) before plating of neurons.

Neurons were prepared from P0-P2 male C57/BL6N mouse cortex. Cortices were dissected out and placed in an enzymatic solution (DMEM plus 0.2 mg/mL L-Cysteine, 1 mM $CaCl_2$, 0.5 mM EDTA) containing papain (22.5 U/mL; Worthington) at 37°C for 1 hr.  Following digestion, the enzymatic solution was replaced with an inactivation solution (DMEM plus 2.5 mg/mL albumin, 2.5 mg/mL trypsin-inhibitor) and incubated again for 5 min at 37°C. Inactivation solution was removed and cortical tissue was triturated with a 200 µL pipet tip until fully dissociated in neuronal growth media, Neural Basal A medium (NBA; Gibco REF 10-888-022) supplemented with 2% B27 (Gibco REF 17-540-044), 1% Glutamax (Gibco REF 35-050-038), and Penicillin/Streptomycin (10 U/mL/1 µg/mL). The cell solution was diluted to a concentration of 250 cells /µL, plated on the pre-treated coverslips in a 200 µL volume held by surface tension, and incubated for 37°C (5% CO2) for 1 hr. After cell adhesion, coverslips containing neurons were transferred to the 6-well astrocyte feeder layer petri dished with premade paraffin posts, pre-filled with neuronal growth media (supplemented NBA) and maintained in a 37°C (5% CO2) incubator. At DIV6, 1 mL of fresh supplemented NBA was added per well. After 14 days *in vitro*, cultures were removed from the incubator, washed with

ice-cold, sterile DPBS and the petri dish was placed directly on dry ice for immediate freezing. Frozen cultures were stored at -80°C until processing for single nuclei RNA sequencing.

*Single Nuclei RNA Sequencing*

Frozen cultures were processed to undergo single-nuclei encapsulation, barcoding, and sequencing library preparation using the Chromium GEM 3' Kit v3.1 from 10X Genomics. In brief, nuclei were resuspended in nuclei wash buffer (PBS; 1% BSA; 0.4U/µl RNAse inhibitor), and 3.5 µl DAPI was added, followed by incubation on ice. The mixture was filtered through a 40 µm Flowmi cell strainer and sorted with a 100 µm nozzle in an Eppendorf tube containing 200 µL sort buffer (PBS; 2% BSA; 2U/µl RNASe inhibitor). Single nuclei libraries were generated according to the Chromium Next GEM Single Cell 3' Reagent Kits v3.1 (Dual Index) user guide (CG0003154) by 10x Genomics. Sequencing of the single nuclei libraries was performed on a NovaSeq X Plus sequencer (Illumina).

Raw reads were processed using Cell Ranger v9.0.1 and aligned to the GRCm39 (2024-A) mouse reference. Ambient RNA and doublets were removed using CellBender (Fleming et al., 2023) and DoubletDetection (Gayoso and Shor, 2022), respectively. Cells were retained based on the following quality thresholds: 700 < n_genes < 6,500, percent_mito < 1%, and n_counts < 40,000, yielding 14,732 cells and 26,253 genes for downstream analysis. All subsequent analysis was performed in SCANPY (Wolf et al., 2018). Dimensionality reduction and batch correction were performed using scVI (Gayoso et al., 2022) on the top 4,000 highly variable genes, and the resulting latent representation was used for UMAP visualization and Leiden clustering (McInnes et al., 2018; Traag et al., 2019).

Subclass labels were transferred from the developmental mouse primary visual cortex taxonomy (Cheng et al., 2022) using the PopV framework. Four independent reference models were constructed from *in vivo* datasets at postnatal days P8, P14, P17, and P21, and *in vitro* cells were mapped onto each reference individually. At subclass level, cells supported by more than five of eight models were classified as higher-agreement, whereas cells supported by five or fewer models were classified as lower-agreement. Annotation confidence was then summarized at the cluster level by calculating the fraction of lower-agreement cells within each Leiden cluster. Clusters containing fewer than 30% lower-agreement cells were classified as high-consensus, whereas clusters exceeding this threshold were classified as enriched for low-consensus. The same hierarchical procedure was applied for type-level annotations using a PopV ensemble of seven annotation models. At type-level, cells supported by more than four of seven models were classified as higher-agreement, and cluster-level consensus was defined analogously based on the proportion of lower-agreement cells within each Leiden cluster.

Transcriptional correspondence between *in vitro* and *in vivo* populations was assessed using two complementary approaches. Marker gene overlap was quantified by computing the Jaccard index between the top 30 subclass marker genes (Wilcoxon rank-sum test) identified independently in the *in vitro* and each reference stage. Pseudobulk Spearman correlations were computed per subclass across stages using the top 4,000 highly variable genes selected from the *in vivo* reference, and the median pairwise correlation was reported per subclass–stage combination.

To quantify transcriptional dispersion, cell to centroid correlation distance was computed for each cell using Decibel (Ibañez-Solé et al., 2022). *In vitro* and P14 *in vivo* cells were concatenated, normalized to 10,000 counts per cell, and log transformed. Genes detected in fewer than 10 cells were excluded.

Pseudobulk differential expression between *in vitro* and *in vivo* cells was performed per subclass using DESeq2 (Love et al., 2014) as implemented in PyDESeq2 (Muzellec et al., 2023), with condition as the design factor. Mitochondrial genes were excluded, and differentially expressed genes were defined by adjusted p-value < 0.05 and $|\log_2 FC| > 1$. Genes upregulated *in vitro* and genes upregulated *in vivo* were tested for Gene Ontology enrichment using Enrichr via GSEApy (Fang et al., 2023), retaining terms with adjusted p-value < 0.05.

## References


Cheng, S., Butrus, S., Tan, L., Xu, R., Sagireddy, S., Trachtenberg, J.T., Shekhar, K. and Zipursky, S.L., 2022. Vision-dependent specification of cell types and function in the developing cortex. Cell, 185(2), pp.311-327.

Fang, Z., Liu, X. and Peltz, G., 2023. GSEApy: a comprehensive package for performing gene set enrichment analysis in Python. Bioinformatics, 39(1), p.btac757.

Fleming, S.J., Chaffin, M.D., Arduini, A., Akkad, A.D., Banks, E., Marioni, J.C., Philippakis, A.A., Ellinor, P.T. and Babadi, M., 2023. Unsupervised removal of systematic background noise from droplet-based single-cell experiments using CellBender. Nature methods, 20(9), pp.1323-1335.

Gayoso, A. and Shor, J., 2022. JonathanShor/doubletdetection: doubletdetection v4. 2. Zenodo https://doi.org/10.5281/ZENODO, 6349517.

Gayoso, A., Lopez, R., Xing, G., Boyeau, P., Valiollah Pour Amiri, V., Hong, J., Wu, K., Jayasuriya, M., Mehlman, E., Langevin, M. and Liu, Y., 2022. A Python library for probabilistic analysis of single-cell omics data. Nature biotechnology, 40(2), pp.163-166.

Ibáñez-Solé, O., Ascensión, A.M., Araúzo-Bravo, M.J. and Izeta, A., 2022. Lack of evidence for increased transcriptional noise in aged tissues. Elife, 11, p.e80380.

Love, M.I., Huber, W. and Anders, S., 2014. Moderated estimation of fold change and dispersion for RNA-seq data with DESeq2. Genome biology, 15(12), p.550.

McInnes, L., Healy, J. and Melville, J., 2018. Umap: Uniform manifold approximation and projection for dimension reduction. arXiv preprint arXiv:1802.03426.

Muzellec, B., Teleńczuk, M., Cabeli, V. and Andreux, M., 2023. PyDESeq2: a python package for bulk RNA-seq differential expression analysis. Bioinformatics, 39(9), p.btad547.

Traag, V.A., Waltman, L. and Van Eck, N.J., 2019. From Louvain to Leiden: guaranteeing well-connected communities. Scientific reports, 9(1), p.5233.

Wolf, F.A., Angerer, P. and Theis, F.J., 2018. SCANPY: large-scale single-cell gene expression data analysis. Genome biology, 19(1), p.15.

**Supplementary Figure S1.**

A

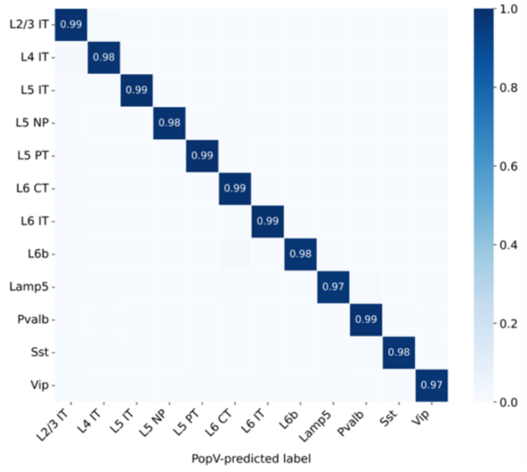


| Label | TPR | F1-Score |
|---|---|---|
| L2/3 IT | 0.986 | 0.981 |
| L4 IT | 0.982 | 0.985 |
| L5 IT | 0.990 | 0.990 |
| L5 NP | 0.981 | 0.976 |
| L5 PT | 0.992 | 0.992 |
| L6 CT | 0.987 | 0.990 |
| L6 IT | 0.991 | 0.993 |
| L6b | 0.976 | 0.976 |
| Lamp5 | 0.970 | 0.968 |
| Pvalb | 0.986 | 0.982 |
| Sst | 0.977 | 0.985 |
| Vip | 0.974 | 0.972 |

B

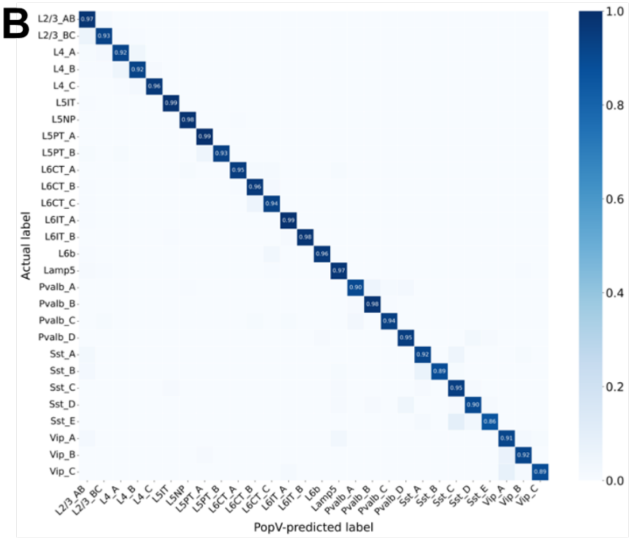


| Label | TPR | F1-Score |
|---|---|---|
| L2/3_AB | 0.974 | 0.962 |
| L2/3_BC | 0.928 | 0.923 |
| L4_A | 0.92 | 0.925 |
| L4_B | 0.924 | 0.937 |
| L4_C | 0.963 | 0.957 |
| L5IT | 0.988 | 0.987 |
| L5NP | 0.981 | 0.985 |
| L5PT_A | 0.993 | 0.987 |
| L5PT_B | 0.935 | 0.956 |
| L6CT_A | 0.946 | 0.921 |
| L6CT_B | 0.965 | 0.966 |
| L6CT_C | 0.936 | 0.938 |
| L6IT_A | 0.987 | 0.988 |
| L6IT_B | 0.98 | 0.982 |
| L6b | 0.961 | 0.978 |
| Lamp5 | 0.97 | 0.956 |
| Pvalb_A | 0.904 | 0.937 |
| Pvalb_B | 0.978 | 0.938 |
| Pvalb_C | 0.938 | 0.933 |
| Pvalb_D | 0.947 | 0.923 |
| Sst_A | 0.921 | 0.929 |
| Sst_B | 0.89 | 0.942 |
| Sst_C | 0.945 | 0.885 |
| Sst_D | 0.903 | 0.915 |
| Sst_E | 0.859 | 0.909 |
| Vip_A | 0.915 | 0.901 |
| Vip_B | 0.92 | 0.914 |
| Vip_C | 0.891 | 0.934 |

C

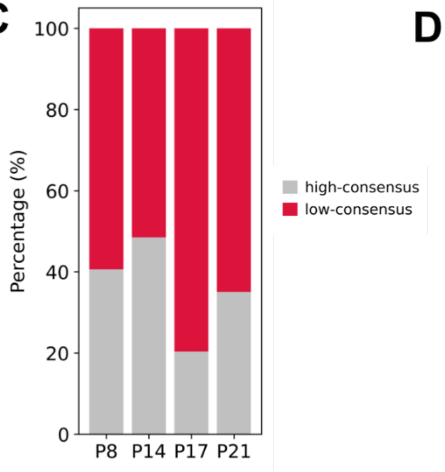


D

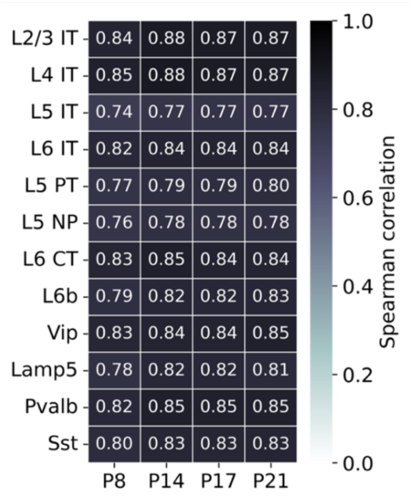

**Supplementary Figure S1**: Global molecular signatures of cell identity are preserved in cultured cortical neurons. A) and B) Row-normalized confusion matrices showing PopV label transfer performance against original reference labels. Each row represents the true reference label and each column the PopV-predicted label. Values along the diagonal indicate the true positive rate (TPR) for each class. Accompanying tables summarize the F1 score and TPR for each class. The upper confusion matrices and tables (A) show performance at the subclass-level, whereas the lower confusion matrices and tables (B) show performance at the type-level. C) Stacked bar plot showing the proportion of cells assigned to high-consensus (grey) or low-consensus (red) Leiden clusters when mapping *in vitro*-derived neurons to postnatal mouse cortical reference atlases (P8, P14, P17, and P21) at type-level. Cell-level agreement was defined by the number of annotation models supporting the majority-vote label. Clusters with fewer than 30% cells supported by five or fewer of eight models were classified as high-consensus. D) Heatmap showing the median Spearman rank correlation between sample-averaged expression profiles of matched neuronal subclasses in the *in vitro* dataset and *in vivo* reference datasets at postnatal stages P8, P14, P17, and P21. Higher values indicate greater transcriptomic similarity.

**Supplementary Figure S2**

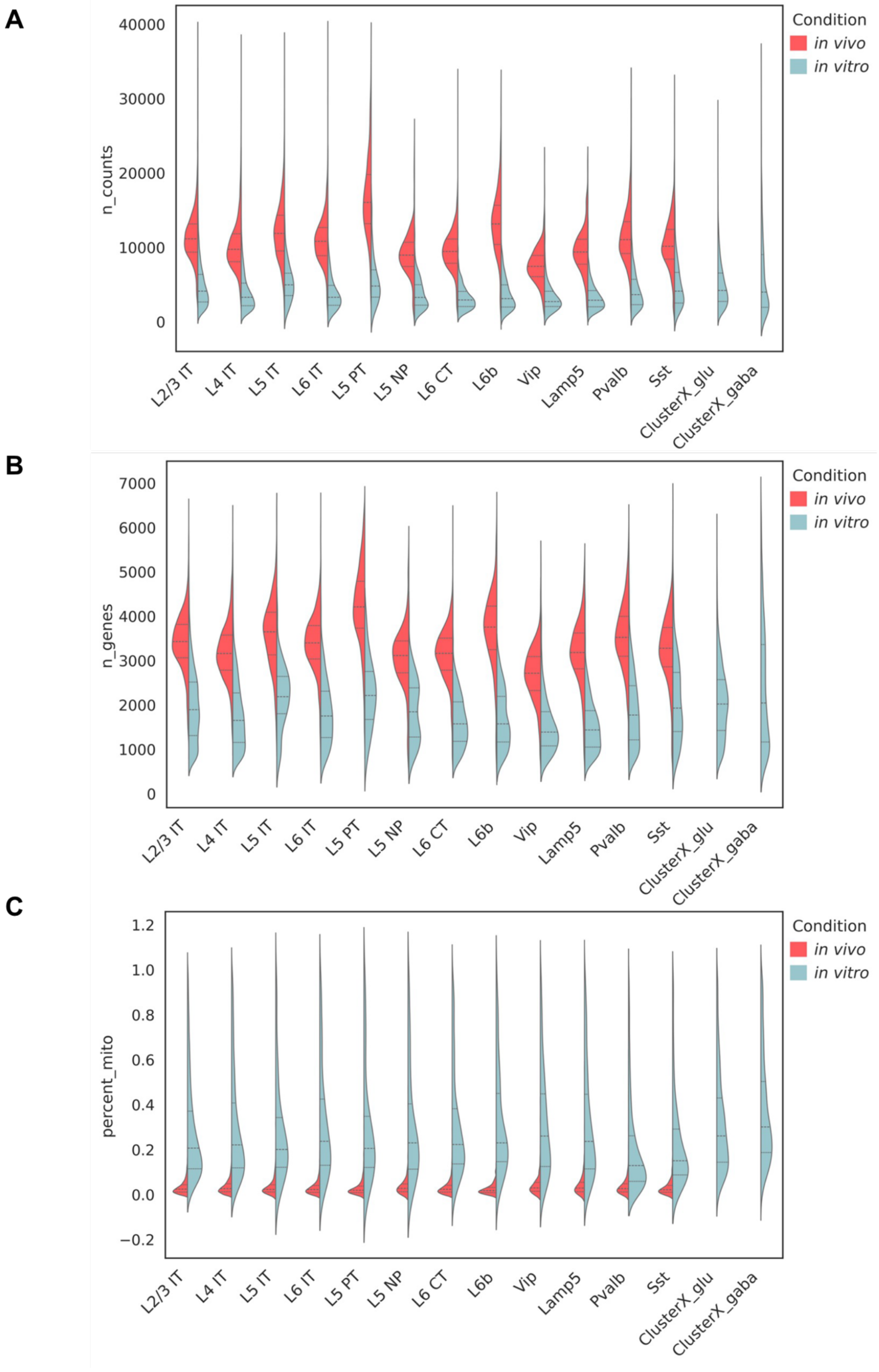

**Supplementary Figure S2:** Comparative quality assessment of *in vitro* and *in vivo* datasets. Split violin plots comparing key quality control (QC) metrics across matched neuronal subclasses. Distributions represent cells from the *in vivo* reference (pink) and the *in vitro* query (teal). Horizontal dashed lines indicate the interquartile range and median within each population. (A) Distribution of the total Unique molecular identifiers (UMI) counts per cell (n_counts). (B) Distribution of the number of unique genes detected per cell (n_genes). (C) Distribution of the percentage of mitochondrial transcript content (percent_mito).

**Supplementary Figure S3**

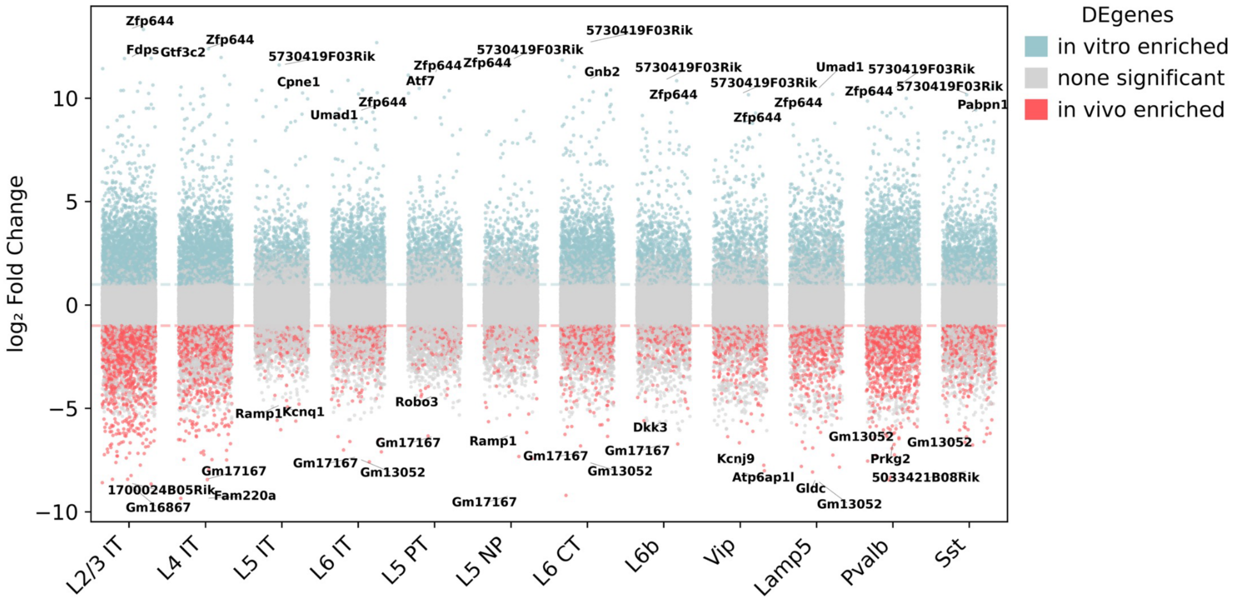


**Supplementary Figure S3:** Divergent transcriptional programs in cultured neurons and the emergence of low-confidence neuronal populations. Stripplot showing differentially expressed genes (DEGs) across neuronal subclasses *in vitro* versus *in vivo*. Each point represents a gene, plotted by subclass (x-axis) and $\log_2$ fold change (y-axis), with colors indicating significant enrichment in in vitro neurons (teal), in vivo neurons (pink), or non-significant changes (grey). Dashed lines mark ±1 $\log_2$ fold change.
